# High Rate of Change of the Foot in Ecuadorian Children: The Need for Proper Shoe Design

**DOI:** 10.3390/children11060749

**Published:** 2024-06-20

**Authors:** Laura Martín-Casado, Inés Palomo-Fernández, Alberto Aldana-Caballero, Ivan Baltasar-Fernandez, Felix Marcos-Tejedor

**Affiliations:** 1 Department of Nursing, Physiotherapy and Occupational Therapy, Faculty of Health Sciences, University of Castilla-La Mancha, 45600 Talavera de la Reina, Toledo, Spain; laura.martincasado@uclm.es (L.M.-C.); alberto.aldana@uclm.es (A.A.-C.); 2 Department of Sport Science, Faculty of Education, Technical University of Ambato, Ambato 180202, Tungurahua, Ecuador; 3 GENUD Toledo Research Group, Faculty of Sport Sciences, University of Castilla-La Mancha, 45071 Toledo, Spain; ivan.baltasar@uclm.es; 4 Department of Physical Activity and Sports Science, Faculty of Health Sciences, University of Castilla-La Mancha, 45600 Talavera de la Reina, Toledo, Spain; 5 Department of Medical Sciences, Faculty of Health Sciences, University of Castilla-La Mancha, 45600 Talavera de la Reina, Toledo, Spain; felix.marcostejedor@uclm.es

**Keywords:** foot morphology, foot development, arch height index, children, 3D foot digitizer

## Abstract

Background: Physiological changes in the foot may be influenced by external factors such as shoe types or demographic parameters, leading to podiatric conditions in adulthood. The aim of this study was to assess the changes in morphological measurements of the feet of boys and girls during childhood and adolescence. Methods: A total of 1678 Ecuadorian children aged 8 to 17 years participated in the study. The length, width, and height of the foot were analyzed using a 3D scanner to obtain the arch height ratio for the diagnosis of pediatric flat foot. Results: Statistical differences were observed for lengths, widths, and perimeters of the foot in boys aged 5 to 15–16 years and girls aged 5 to 12–13 years. Differences in the height of the navicular bone were found in all age groups, with changes from 2.8 to 4.1% in boys and from 1.3 to 1.5% in girls. The greatest differences between boys and girls of the same age were found at 14 years old onwards. The highest prevalence of flat foot was found in 8-year-old girls (64.9%) and in 12-year-old boys (82.5%). Conclusions: The feet of Ecuadorian children develop progressively during childhood and adolescence. Boys presented with longer and wider feet than girls of the same age. The highest prevalence of flat foot was found in 12-year-old boys and 8-year-old girls.

## 1. Introduction

The foot experiences changes from childhood to adolescence, bearing great mechanical stress that leads to continuous morphological and functional variations that affect its development [1]; therefore, the type of shoe used during this time is of paramount importance [2].

Previous studies focused on analyzing the morphology of pediatric feet and the specific shoes and their influence on the development of the foot, highlighting the importance of recognizing that although the foot of a child presents similar characteristics to that of an adult, it should not be managed equally [2,3,4]. However, shoe manufacturers continue using traditional adult criteria when creating shoe shapes for children, merely scaling to a smaller dimension and considering the lengths to determine the shoe sizes [1].

The development of the pediatric foot is fast-paced. Up to 50% of the total length of the foot is obtained between 12 to 18 months [5], and children aged 3 to 4 present the highest prevalence of flat foot [6]. However, it is not until adolescence that the foot shows a similar appearance to that of an adult and can be determined to be completely formed [7]. Nonetheless, the morphological development of the foot not only depends on the growth rate but also can be determined by other factors such as sex and other demographical parameters. The foot grows in length and width up to 14 to 15 years of age in boys [8] and up to 12 years in girls, with girls showing smaller feet compared to boys of the same age [9]. Furthermore, Mauch et al. [10] observed that German children have flatter and longer feet compared to their Australian counterparts, and Sacco et al. [11] observed that between the ages of 5 and 10, Brazilian children present a narrower forefoot area compared to German children.

In recent years, shoe design has become especially relevant for children to respect the normal development of the foot [2,4,12], but this is only achieved by maintaining a correct relationship between the internal area of the shoe and the length, width, and height of the foot [13]. A poor fit can cause fatigue, falls, problems, and other foot conditions such as hallux valgus, claw-like toes, or hammertoes [1,2,14,15]. Ecuador generally acquires shoe lasts from specialized international suppliers. However, each population has specific needs. Footwear design based on specific growth data and the foot shape of these populations can help prevent podiatric problems. With lasts and designs adapted to local needs, Ecuadorian companies that design and manufacture children’s shoes can innovate and differentiate themselves in the market, offering products that are not only more comfortable but also aligned with the health and well-being of their users.

Lately, 3D scanning has gained momentum in obtaining the morphological measurements of the foot [9,16,17]; however, these studies are centered on European [1,17] or Asian populations [9,18,19]. To our knowledge, no previous studies have analyzed the changes in the feet of Latin American children using this approach. Thus, the aim of this study was to analyze the variations that occur in the morphological measurements of the feet of Ecuadorian boys and girls during childhood and adolescence.

## 2. Materials and Methods

### 2.1. Participants

A total of 1678 children from different provinces of Ecuador (834 boys and 844 girls), aged 5 to 17 years, with an average weight of 34.1 ± 13.2 Kg and an average height of 1.35 ± 0.19 m participated voluntarily in the study (Table 1). All Ecuadorian school-aged children were included, while those with foot abnormalities or malformations, and those who had been treated with foot orthoses or surgically for these conditions, were excluded.

Parents or legal guardians provided written informed consent to participation in the study, which followed the principles of the Declaration of Helsinki and all other applicable laws for data protection. The study was approved by the Bioethical Committee of the University of San Francisco de Quito (2016-083E).

### 2.2. Measurements

All foot measurement data were collected throughout the year 2016.

Participants were weighed and measured using a standing scale and height rod model 420KLWA (Welch Allyn, New York, NY, USA). Measurements to evaluate the structure of both feet were conducted using a device with 8 cameras (INFOOT, I-Ware Laboratory Co., Ltd., Osaka, Japan) [20], which placed the following anatomical landmarks: (1) navicular, (2) the most lateral point of the medial malleolus, (3) the most lateral point of the lateral malleolus, (4) metatarsal tibiale, (5) metatarsal fibulare, (6) first toe joint, (7) fifth toe joint, (8) heel born point, (9) head of 1st metatarsal, (10) head of 2nd metatarsal, (11) cuneiform, and (12) tuberosity of the fifth metatarsal

**Figure 1:**
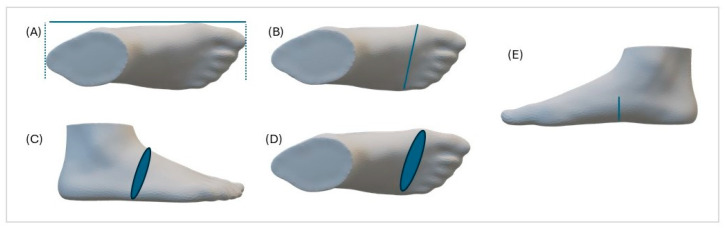
Variables of foot dimensions obtained with 3D scanner: foot length (**A**), metatarsal width (**B**), instep circumference (**C**), metatarsal circumference (**D**), and navicular height (**E**).

All participants were seated on a chair, distributing their weight evenly between both feet, looking straight ahead with their hands holding onto the handle of the scanner. In this position, the following foot anthropometric measurements were taken: foot length (=FL: distance between the most proximal point of the heel and the most distal point of the phalanges); metatarsal width (=MW: distance between the most medial point of the first metatarsal head and the most lateral point of the fifth metatarsal head); metatarsal circumference (=MC: maximal circumference obtained around the metatarsal heads between the first and the fifth); instep circumference (=IC: maximal circumference around the highest point of the cuneiform bones); navicular height (=NH: height from the most prominent point of the plantar arch towards the ground) (Figure 1).

Additionally, the arch height ratio was calculated (=AHR: NH × 100/FL), with an optimal score of ≤0.195 for the diagnosis of pediatric flat foot [21].

### 2.3. Statistical Analysis

Descriptive data are shown as mean ± standard deviation (SD) for continuous variables, whereas categorical variables are expressed as frequencies (*n*) with corresponding percentages (%). The normality of the distribution was examined using Shapiro–Wilk tests, while the homoscedasticity was evaluated using Levene’s test.

Segmented stepwise linear regression analyses were used to examine the relationship between age and foot measurements in boys and girls [22]. An iterative approach was used to detect the potential age points at which a change in slope occurred (6, 7, 8, 9, 10, 11, 12, 13, 14, 15, 16) between 5 and 17 years. Only age points exhibiting a significant change in the slope were incorporated into the final regression model. The slopes obtained from the regression analyses represent the annual rate of change in the study variables. Differences in regression slopes (i.e., annual rate of changes) between boys and girls were evaluated by a comparison of 95% confidence intervals. A mixed-model ANOVA with two fixed effects (sex and age) was used for assessing differences in foot measurements between boys and girls across different ages (from 6 to 17 years). Pairwise comparisons were conducted using Bonferroni’s post hoc tests.

Finally, differences in the prevalence of flat foot between boys and girls across all ages were assessed using the χ^2^ test. All statistical analyses were performed using SPSS v21 (SPSS Inc., Chicago, IL, USA), and the significance level was set at α = 0.05.

## 3. Results

No statistical differences were observed in most of the variables between the left and right foot. Therefore, the mean values of both feet were analyzed for each participant’s foot structure [10].

Table 2 shows the annual rate of changes in various dimensions of the foot in boys and girls. Regarding FL, a significant annual increase of 3.3% to 4.7% was observed in boys between 5 and 15 years (*p* < 0.05) and 3.8% to 5% in girls aged 5 to 12 years (*p* < 0.05), showing significant differences between boys and girls aged 5 to 15 years (*p* < 0.05). Both MW and MC increased from 5 to 16 years in boys (*p* < 0.05) and from 5 to 13 years in girls (*p* < 0.05), with annual changes ranging from 2.7% to 3.8% and from 3.0% to 3.8%, respectively. Differences between sexes were observed from 14 years old onward (*p* < 0.05). IC increased in boys from 5 to 15 years at an annual rate ranging from 2.9% to 3.8% (*p* < 0.05) and in girls from 5 to 13 years at an annual rate ranging from 3.0% to 3.8% (*p* < 0.05), showing significant differences in the annual rate of change between sexes at ages 14 and 15 (*p* < 0.05). Lastly, NH annually increased from 5 to 17 years by 2.8% to 4.1% in boys and 1.3% to 1.5% in girls (*p* < 0.05), showing significant differences between sexes across all ages (*p* < 0.05).

**Figure 2:**
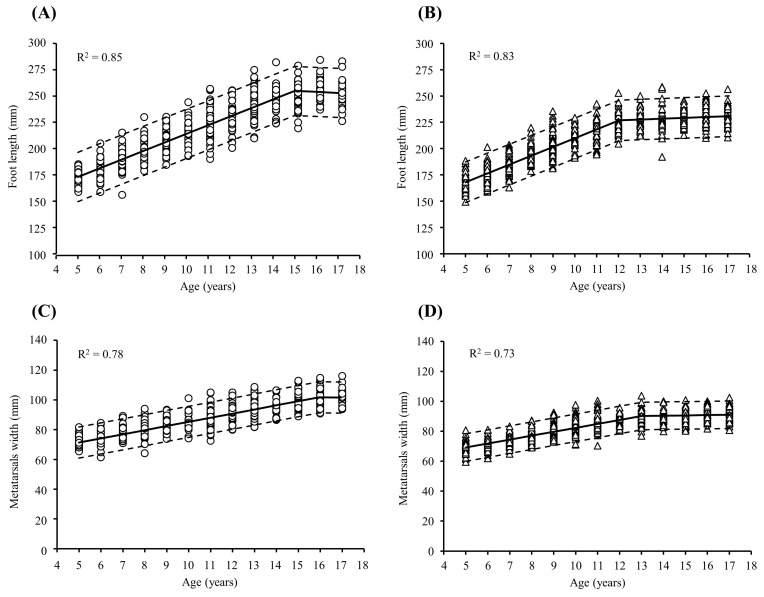
Cross-sectional trajectories of foot length (**A**,**B**) and metatarsal width (**C**,**D**) throughout childhood and adolescence in boys (open circles) and girls (open triangles). Regression lines (continuous lines), 95% confidence intervals (dashed lines), and coefficient of determination (R^2^) values were obtained by segmented regression analysis.

Segmented regression analyses revealed that foot length increased in boys and girls until the ages of 15 (Figure 2A) and 12 years (Figure 2B), respectively, with a flattening of the slope thereafter. Metatarsal width increased in boys and girls until the ages of 16 (Figure 2C) and 13 years (Figure 2D), respectively, with a flattening of the slope thereafter.

**Figure 3:**
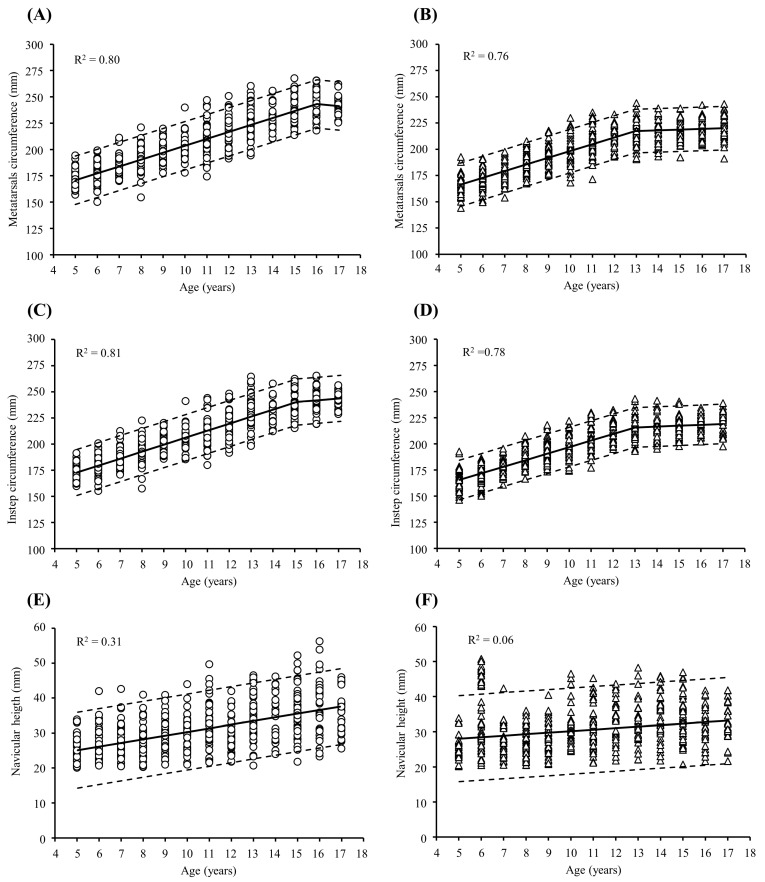
Cross-sectional trajectories of metatarsal circumference (**A**,**B**), instep circumference (**C**,**D**), and navicular height (**E**,**F**) throughout childhood and adolescence in boys (open circles) and girls (open triangles). Regression lines (continuous lines), 95% confidence intervals (dashed lines), and coefficient of determination (R^2^) values were obtained by segmented regression analysis.

In the same line, metatarsal circumference increased in boys and girls until the ages of 16 (Figure 3A) and 13 years (Figure 3B), respectively, while instep circumference increased in boys and girls until the ages of 15 (Figure 3C) and 13 years (Figure 3D), respectively. In both sexes, regarding those variables, a flattening of the slope occurred after those ages; however, regarding the navicular height, no changes in the slope were observed in either boys (Figure 3E) or girls (Figure 3F) from 5 to 17 years.

When comparing the average foot dimensions of boys and girls of the same age, it was observed that boys exhibited greater foot length, metatarsal width, and metatarsal circumference than girls between the ages of 5 and 9 years and between 12 and 17 years (Table 3). However, they presented similar average values for these variables between the ages of 10 and 11 years. Regarding instep circumference, boys exhibited higher average values than girls at all ages (Table 3). Concerning the average values of the navicular height, it was observed that girls had greater navicular height than boys at 6 and 12 years old, with boys’ navicular height surpassing that of girls only from 15 years onwards (Table 3).

**Figure 4:**
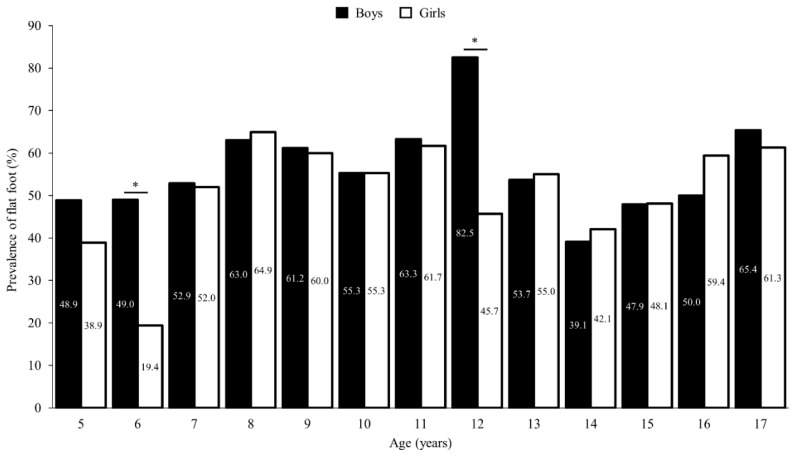
Prevalence of flat foot among boys (black bars) and girls (open bars) by age. * Significant differences between boys and girls of the same age (*p* < 0.05).

Lastly, the highest prevalence of flat foot was observed at 8 years old in girls (64.9%) and at 12 years old in boys (82.5%). The lowest prevalence was observed in 6-year-old girls (19.4%) and in 15-year-old boys (47.9%) (Figure 4). The prevalence of flatfoot was higher in boys aged 6 and 12 years than in girls of the same age (both *p* < 0.05).

## 4. Discussion

The results derived from the present study show, for the first time, differences in the morphology of the foot in Ecuadorian children and adolescents, in relation to sex and age. And even though we are aware that environmental factors may influence these changes, we consider the findings to be of great clinical value and potentially applicable to other population groups.

In boys, foot length increases progressively between the ages of 5 and 15 years (*p* < 0.05), while in girls, this growth occurs from 5 to 12 years of age (*p* < 0.05). This growth pattern is linear (Figure 2) and strongly correlated with height (r = 0.91 for boys and r = 0.87 for girls, *p* < 0.001). Beyond these ages, no significant differences in foot length are observed. The greatest changes occurred from 5 to 6 years in both boys (4.7%) and girls (5.0%) (Table 2). Furthermore, significant differences were observed between boys and girls from 5 to 15 (*p* < 0.05), except between 10 and 11 years, during which no differences between sexes are observed (Table 3). These results concur with previous studies that determined that the most rapid development of the foot occurs at the age of 6 and that the rate of change slows down earlier in girls than in boys [3,16,17], with periods during which boys and girls show no differences to each other [2,17]. With the onset of puberty in girls, foot growth accelerates, reducing the differences with boys. From the age of 12 years, when puberty begins in boys, differences are observed once again. Our results indicate that the feet of Ecuadorian children grow until the age of 16 years in boys and 13 years in girls, while other studies suggest that the development of the feet ends between the ages of 11 and 12 years in the case of European children [17] or between 13 and 14 in the case of Asian children [3], in boys and girls. These differences could be caused by the intrinsic characteristics of each studied population. However, although the growth pattern may differ between boys and girls based on their demographic situation, the relationship between the development of FL and the onset of puberty is generally consistent [3,23,24].

In the present study, significant changes were observed in MW (boys: 3.8–2.7%/year; girls: 3.8–3.0%/year; both *p* < 0.05), MC (boys: 3.8–2.9%/year; girls: 3.8–3.0% year; both *p* < 0.05), and IC (boys: 3.8–2.8%/year; girls: 3.9–3.0%/year; both *p* < 0.05) from 5 to 15 years in boys and from 5 to 13 years in girls (Figure 3), showing higher values in boys’ feet compared to girls’ feet of the same age (Table 2). The differences between boys and girls are evident from 14 years old onwards (*p* < 0.05) (Table 3). The anatomy of the forefoot is especially vulnerable to the adjustment of shoes. Some authors have suggested a correlation between narrow shoes and forefoot malformations [2,25]. Additionally, it is known that the width of the metatarsals is a predisposing factor for hallux valgus [26]. The high rate of change in the width and perimeter of the forefoot requires special attention from children’s shoe manufacturers, which generally do not consider the anthropometric differences associated with digital growth patterns [1,2]. This is particularly relevant in older children, who are more influenced by the fashion industry and the use of pointy shoes, putting the foot at risk.

The NH showed significant differences for all groups, with increases of 1.5%/year in girls and up to 4%/year in boys at early ages, from 5 to 6 years (Table 2). We found a significant correlation between BMI and the arch height ratio in girls (r = −0.12, *p* = 0.005) and a trend in boys (r = −0.08, *p* = 0.067). However, it is important to note that this correlation was low, considering the Pearson correlation coefficient value. This might suggest that weight could be influencing the development of flat foot in children, but other factors such as joint hypermobility or shoe shape might have a greater impact on the development of flat foot, as reported in previous studies [27]. The plantar arch begins to develop progressively from the onset of weight-bearing standing positions in children and continues for the next 10 years. Our results are in line with other studies that have described lower plantar arches as common in the first few years of childhood [14,28,29]. Nonetheless, the highest prevalence of flat foot was found at the age of 12 in boys (82.5%) and at the age of 8 in girls (64.9%), with significant differences between groups at the ages of 6 and 12 years (*p* < 0.05) (Figure 4). This is especially relevant for podiatrists and pediatricians, as the possibility for correction decreases exponentially after this age. Periodic follow-ups and muscle strengthening of the foot during the first years of development could prevent the collapse of the plantar arch, which could result in future problems in adulthood.

Considering these results, manufacturers should take into account the morphological changes of the foot when designing shoes for children, which are not normally considered [3,10]. It would be beneficial for children’s shoes to accommodate the various developmental changes, providing sufficient space in length and width in the forefoot according to the age of the child. Additionally, they should avoid including anatomical elements on the inside that could interfere with the natural development of the plantar arch. Taking these space requirements into consideration in footwear design, better adaptation to the natural growth of the foot could be achieved, thereby preventing deformities such as claw toes, hallux valgus, or flat foot in adulthood.

## 5. Conclusions

The feet of Ecuadorian children progressively grow from the ages of 5 to 15 years in boys and from 5 to 12 years in girls, with annual changes of up to 4.7–5.0% in length and 3.8–3.9% in width. Boys generally exhibit longer and wider feet compared to girls of the same age. The plantar arch shows an increase of 1.5%/year in girls and up to 4%/year in boys from 5 to 6 years old. The highest prevalence of flat foot is observed at the age of 8 years in girls (64.9%) and at the age of 12 years in boys (82.5%). These results provide valuable insights for improving the design of children’s shoes.

## Figures and Tables

**Table 1:** Descriptive characteristics of the study participants.

Age (Years)	Weight (kg)		Height (m)		BIM (kg/m^2^)	
	Boys	Girls	Boys	Girls	Boys	Girls
5	18.36 ± 1.42	187.97 ± 1.80	1.09 ± 0.04	1.07 ± 0.05	15.54 ± 0.91	15.71 ± 0.81
6	20.71 ± 2.38	19.44 ± 2.04	1.14 ± 0.05	1.13 ± 0.05	15.88 ± 1.37	15.32 ± 1.14
7	22.47 ± 2.91	22.28 ± 2.56	1.19 ± 0.05	1.19 ± 0.05	15.83 ± 1.54	15.77 ± 1.13
8	25.01 ± 3.06	24.58 ± 2.56	1.24 ± 0.05	1.23 ± 0.04	16.19 ± 1.18	16.22 ± 1.23
9	28.16 ± 3.19	27.91 ± 3.58	1.29 ± 0.05	1.28 ± 0.06	16.92 ± 1.13	17.01 ± 1.20
10	30.63 ± 4.24	29.53 ± 5.13	1.33 ± 0.06	1.34 ± 0.06	17.24 ± 1.32	16.46 ± 2.18
11	33.05 ± 5.60	33.38 ± 5.18	1.37 ± 0.07	1.38 ± 0.07	17.41 ±1.89	17.44 ± 1.63
12	37.73 ± 5.70	39.67 ± 5.28	1.43 ± 0.08	1.46 ± 0.08	18.42 ± 1.59	18.65 ± 1.44
13	43.90 ± 7.43	43.30 ± 5.32	1.52 ± 0.08	1.50 ± 0.06	18.96 ±1.77	19.34 ± 1.79
14	44.67 ± 7.18	46.61 ± 7.24	1.54 ± 0.09	1.51 ± 0.08	18.71 ±1.73	20.42 ± 1.96
15	51.87 ± 8.99	48.77 ± 5.13	1.61 ± 0.11	1.53 ± 0.05	19.74 ± 2.08	20.82 ± 1.70
16	56.20 ± 7.13	50.79 ± 5.33	1.66 ± 0.06	1.55 ± 0.07	20.22 ± 1.77	21.20 ± 1.89
17	56.97 ± 6.49	52.25 ± 5.32	1.63 ± 0.06	1.55 ± 0.06	21.28 ± 1.86	21.85 ± 1.93

The values are presented as mean ± standard deviation (SD).

**Table 2:** Average annual percentage differences (% year^−1^) in foot measurements by different age groups, for boys and girls.

Age (Years)	Foot Length (mm)		Metatarsal Width (mm)		Metatarsal Circumference (mm)		Instep Circumference (mm)		Navicular Height (mm)	
	Boys	Girls	Boys	Girls	Boys	Girls	Boys	Girls	Boys	Girls
5	−	−	−	−	−	−	−	−	−	−
6	**4.7**	**5.0** *	**3.8**	**3.8**	**3.8**	**3.9**	**3.8**	**3.8**	**4.1**	**1.5** *
7	**4.5**	**4.7** *	**3.7**	**3.6**	**3.7**	**3.7**	**3.7**	**3.6**	**3.9**	**1.5** *
8	**4.3**	**4.5** *	**3.5**	**3.5**	**3.6**	**3.6**	**3.6**	**3.5**	**3.8**	**1.5** *
9	**4.1**	**4.3** *	**3.4**	**3.4**	**3.4**	**3.5**	**3.4**	**3.4**	**3.6**	**1.4** *
10	**3.9**	**4.1** *	**3.3**	**3.3**	**3.3**	**3.3**	**3.3**	**3.3**	**3.5**	**1.4** *
11	**3.8**	**4.0** *	**3.2**	**3.2**	**3.2**	**3.2**	**3.2**	**3.2**	**3.4**	**1.4** *
12	**3.6**	**3.8** *	**3.1**	**3.1**	**3.1**	**3.1**	**3.1**	**3.1**	**3.3**	**1.4** *
13	**3.5**	0.4 *	**3.0**	**3.0**	**3.0**	**3.0**	**3.0**	**3.0**	**3.2**	**1.4** *
14	**3.4**	0.3 *	**2.9**	0.2 *	**2.9**	0.3 *	**2.9**	0.4 *	**3.1**	**1.4** *
15	**3.3**	0.3 *	**2.8**	0.2 *	**2.9**	0.3 *	**2.9**	0.4 *	**3.0**	**1.3** *
16	−0.4	0.3	**2.7**	0.2	**2.8**	0.3 *	0.7	0.4	**2.9**	**1.3** *
17	−0.4	0.3	−0.2	0.2	−0.8	0.3	0.7	0.4	**2.8**	**1.3** *

Bold values indicate significant differences compared to a slope equal to zero (*p* < 0.05). * Significantly different compared to boys of the same age (*p* < 0.05).

**Table 3:** Foot measurements for boys and girls, from 5 to 17 years old (displayed as mean ± standard).

Age (Years)	Foot Length (mm)		Metatarsal Width (mm)		Metatarsal Circumference (mm)		Instep Circumference (mm)		Navicular Height (mm)	
	Boys	Girls	Boys	Girls	Boys	Girls	Boys	Girls	Boys	Girls
5	172.4 ± 7.2	167.7 ± 9.4 *	72.1 ± 3.8	69.3 ± 4.4 *	172.6 ± 7.9	166.1 ± 9.9 *	173.5 ± 7.4	166.1 ± 10.1 *	25.6 ± 3.5	25.3 ± 3.2
6	180.1 ± 8.9	176.2 ± 8.8 *	74.3 ± 4.3	71.2 ± 3.7 *	177.6 ± 9.8	171.2 ± 8.5 *	180.4 ± 10.1	170.8 ± 7.9 *	26.8 ± 4.2	34.3 ± 9.5 *
7	191.3 ± 8.8	185.3 ± 9.4 *	77.7 ± 4.1	74.1 ± 3.8 *	185.6 ± 9.1	177.3 ± 8.8 *	186.8 ± 8.4	178.1 ± 8.0*	27.3 ± 4.7	27.0 ± 4.1
8	197.4 ± 10.1	196.4 ± 8.5 *	80.0 ± 5.0	77.8 ± 4.5 *	191.4 ± 11.3	186.5 ± 9.9 *	193.4 ± 11.2	187.7 ± 8.7*	27.3 ± 4.3	27.2 ± 4.0
9	208.3 ± 10.2	204.0 ± 11.5 *	83.2 ± 4.3	80.3 ± 4.7 *	198.9 ± 9.8	192.7 ± 10.8 *	200.9 ± 8.7	193.2 ± 9.4 *	29.5 ± 4.7	28.3 ± 4.2
10	214.1 ± 11.2	212.6 ± 9.0	84.8 ± 4.9	83.5 ± 5.8	203.1 ± 11.2	199.5 ± 13.6	206.0 ± 10.6	198.3 ± 11.6 *	29.7 ± 5.0	31.0 ± 5.5
11	223.1 ± 14.7	219.2 ± 10.5	87.5 ± 6.8	86.2 ± 5.9	208.7 ± 15.0	206.9 ± 13.4	211.2 ± 13.5	205.0 ± 11.9 *	31.8 ± 6.0	30.9 ± 5.8
12	231.2 ± 13.5	225.6 ± 10.8 *	91.0 ± 6.2	87.8 ± 4.0 *	218.0 ± 14.1	211.7 ± 9.7 *	219.2 ± 13.9	211.5 ± 9.7 *	29.4 ± 4.7	32.6 ± 5.6 *
13	241.9 ± 13.5	227.3 ± 9.2 *	95.4 ± 6.2	89.6 ± 5.4 *	228.6 ± 14.5	215.4 ± 11.5 *	230.5 ± 13.9	215.1 ± 10.9 *	34.6 ± 6.0	32.6 ± 5.9
14	246.2 ± 13.4	227.8 ± 13.0 *	95.2 ± 5.9	89.6 ± 5.1 *	229.3 ± 13.0	216.1 ± 11.7 *	231.2 ± 11.9	214.8 ± 11.0 *	35.1 ± 6.6	34.3 ± 6.2
15	249.2 ± 16.5	230.2 ± 7.5 *	99.0 ± 5.7	90.0 ± 4.1 *	237.7 ± 12.4	216.8 ± 9.2 *	238.7 ± 12.5	217.5 ± 8.4 *	36.1 ± 6.6	33.3 ± 6.0 *
16	255.7 ± 10.0	232.6 ± 10.5 *	101.8 ± 6.3	91.2 ± 4.5 *	242.8 ± 12.9	219.9 ± 9.3 *	243.6 ± 11.5	220.0 ± 7.9 *	37.6 ± 8.1	32.0 ± 5.5 *
17	251.6 ± 12.4	230.9 ± 9.0 *	101.8 ± 4.2	91.9 ± 4.9 *	242.3 ± 8.8	221.3 ± 11.3 *	242.3 ± 8.2	220.6 ± 10.3 *	35.8 ± 6.0	32.4 ± 5.5 *

* Significantly different compared to boys of the same age (*p* < 0.05).

## Data Availability

The original contributions presented in the study are included in the article, further inquiries can be directed to the corresponding author.
